# Development of a new toolbox for mouse PET–CT brain image analysis fully based on CT images and validation in a PD mouse model

**DOI:** 10.1038/s41598-022-19872-4

**Published:** 2022-09-22

**Authors:** L. Presotto, V. Bettinardi, D. Mercatelli, M. Picchio, M. Morari, R. M. Moresco, Sara Belloli

**Affiliations:** 1 Nuclear Medicine Department, IRCCS San Raffaele Scientific Institute, Milan, Italy; 2 Milan Centre for Neuroscience, University of Milano - Bicocca, Milan, Italy; 3 Department of Neuroscience and Rehabilitation, University of Ferrara, Ferrara, Italy; 4 Vita-Salute San Raffaele University, Milan, Italy; 5 Medicine and Surgery Department, University of Milano - Bicocca, Monza, MB Italy; 6 Institute of Molecular Bioimaging and Physiology (IBFM) of National Research Council (CNR), Segrate, MI Italy

**Keywords:** Computational biology and bioinformatics, Data processing

## Abstract

Automatic analysis toolboxes are popular in brain image analysis, both in clinical and in preclinical practices. In this regard, we proposed a new toolbox for mouse PET–CT brain image analysis including a new Statistical Parametric Mapping-based template and a pipeline for image registration of PET–CT images based on CT images. The new templates is compatible with the common coordinate framework (CCFv3) of the Allen Reference Atlas (ARA) while the CT based registration step allows to facilitate the analysis of mouse PET–CT brain images. From the ARA template, we identified 27 volumes of interest that are relevant for in vivo imaging studies and provided binary atlas to describe them. We acquired 20 C57BL/6 mice with [^18^F]FDG PET–CT, and 12 of them underwent 3D T2-weighted high-resolution MR scans. All images were elastically registered to the ARA atlas and then averaged. High-resolution MR images were used to validate a CT-based registration pipeline. The resulting method was applied to a mouse model of Parkinson’s disease subjected to a test–retest study (n = 6) with the TSPO-specific radioligand [^18^F]VC701. The identification of regions of microglia/macrophage activation was performed in comparison to the Ma and Mirrione template. The new toolbox identified 11 (6 after false discovery rate adjustment, FDR) brain sub-areas of significant [^18^F]VC701 uptake increase versus the 4 (3 after FDR) macro-regions identified by the Ma and Mirrione template. Moreover, these 11 areas are functionally connected as found by applying the Mouse Connectivity tool of ARA. In conclusion, we developed a mouse brain atlas tool optimized for PET–CT imaging analysis that does not require MR. This tool conforms to the CCFv3 of ARA and could be applied to the analysis of mouse brain disease models.

## Introduction

The definition of the brain anatomical spaces, where structures and regions are precisely localized, is at the basis of the study of brain functionality and connectivity across different species. Brain atlases define the organization of anatomical and functional regions in a three-dimensional (3D) volume. Stereotaxic brain atlases are mostly used in neurological studies and are drawn on Magnetic Resonance (MR) images where voxels are grouped into anatomical structures. Mouse and rat represent mammalian models of brain development and several rodent atlases are available based on different strategies, i.e. haematoxylin and eosin, Nissl staining^1^ (https://www.hms.harvard.edu/research/brain/intro.html) or density, position and organization of cells^2^. The most representative and popular atlases are those developed by Franklin, Paxinos and Watson for mouse and rat^3,4^ and by the Allen Institute for Brain Sciences with the more recent Allen Reference Atlas (ARA) for the mouse brain (https://atlas.brain-map.org/). Besides the differences in anatomical structures borders and ontology^5^, the major difference between the Franklin-Paxinos and the ARA atlases is the definition of the 3D coordinates within the brain volume. The former is considered the reference atlas for stereotaxic surgery and is based on a classical neuroanatomical approach with Bregma as zero point. The latter has been developed more recently by integrating different types of information from in vivo imaging (i.e. Positron Emission Tomography-PET), MR and post-mortem imaging (two-photon tomography, histology) with genome-wide image database and gene expression data^6,7^. The ARA has higher spatial resolution compared to other atlases (10 µm isotropic voxel) but the relation of the template with anatomical landmarks as Bregma is unknown. Therefore, a spatial alignment with high resolution images as MR is necessary to validate stereotaxic coordinates^8^.

PET is a functional molecular imaging technique that leverages on specific radiotracers to provide information about the distribution of a specific target, e.g. a receptor, enzyme or process, within brain or peripheral areas in pathological compared to physiological conditions^9^. Anatomical information is obtained through the integration of PET imaging with different modalities as Computed Tomography (CT) and MR. PET-MR hybrid devices are a recent development for both clinical and preclinical purposes. Technical considerations and the high costs limit the diffusion of this equipment and to date the most common solution is represented by PET–CT systems^10^. This combination allows the localization of the molecular target in the anatomical space, even if the reference technique for the brain anatomy is MR imaging^11^. Therefore, the identification and quantification of radioligand uptake areas within PET image volume is obtained by applying structural MR-derived templates that segmented the brain into anatomical/functional regions. Typically, the workflow consists in a sequence of image alignment and deformations to a reference space that make it possible to compare different subjects or the same subject in different conditions as in longitudinal studies^12^.

The MR templates included in most software used for PET images analysis (e.g. https://www.pmod.com/web/; https://www.fil.ion.ucl.ac.uk/spm/; https://mipav.cit.nih.gov/) account for non-deformable and pre-defined macro-regions. This entails some limitations in the identification of functional sub-areas that could be characterized by ligand uptake modification, also influencing the correlation analysis across the different areas. Another limitation of MR-based templates is that they work most efficiently when a high-resolution 3D scan is present, which is not always the case in murine studies.

In the present work, we built a brain template compatible with Statistical Parametric Mapping (SPM) software, which exploit the ARA volume for the identification of brain structures using the same ontology and coordinate framework. In this way, the areas where a modification of a biomarker is highlighted by PET could be matched with the additional information provided by ARA, such as in situ hybridization, cell projection maps and in vitro cell characterization. The gold standard procedure to achieve optimal spatial registration is based on high resolution isotropic 3D MR. The acquisition of such an image however is lengthy, costly, and it might not even be always feasible, as performing two long procedures requiring anaesthesia in the same day on the same animal can be unethical. Therefore, to better exploit this template, we also introduced and validated a pipeline that allows transforming individual images to this standard space using a CT image and not an MR sequence. This makes the analysis more versatile, applicable to a wide range of PET tracers and independent on the acquisition, on a different scanner, of a high-resolution 3D MR image. As the analysis does not depend on the availability of a tracer-specific template, it is immediately widely applicable. Furthermore, its performance does not depend on the contrast in the PET image, which can vary depending on the tracer target. Indeed, compared to the PET-template based one, the CT-based spatial normalization is unbiased^13^.

In this work, after a detailed description and validation of the proposed method, we provided an example application to the analysis of a Parkinson’s disease (PD) model.

## Materials and methods

### Animals and imaging

Experimental procedures involving the use of animals complied with the EU Directive 2010/63/EU for animal experiments and were approved by the Ethical Committees of the San Raffaele Research Institute (Milan) and the Italian Ministry of Health (license n. 237/2019-PR). The study was carried out in compliance with the ARRIVE guidelines.

### Study design

To optimize and build the pipeline of PET–CT template creation, we acquired twenty adult C57BL/6 male mice with PET and the radioligand for glucose metabolism 2-Deoxy-2-[^18^F]-fluoroglucose ([^18^F]FDG), and CT. Twelve of them were also acquired with a 3D T2-weighted high resolution MR sequence.

The template analysis was then applied to the study of microglia/macrophage activation in a PD mouse model obtained with the subacute administration of the neurotoxin 1-methyl-4-phenyl-1,2,5,6-tetrahydropyridine (MPTP)^14^. Six mice were acquired with the TSPO specific radiotracer [^18^F]VC701^15^ before and after neurotoxin treatment and analysed using the new brain atlas template in comparison to PMOD 4.1 (PMOD TECHNOLOGIES LLC, Zürich, Switzerland) VOI template^12^.

### Animal images acquisition and reconstruction for brain template

Three-month-old C57BL/6JRj male mice (n = 20) were purchased from Janvier Labs (Paris, FR). The radiotracer [^18^F]FDG was prepared for clinical use following European Pharmacopeia VIII Edition and i.v. injected in animals in fasting conditions (mean injected activity: 4.5 ± 0.3 MBq).

CT and PET acquisitions were performed 60 min post injection using X-Cube^®^ and β-Cube^®^ (Molecubes, Gent, BE), respectively. Each animal was anesthetized with 2% isoflurane in medical air and then positioned prone on the X-Cube scanner bed for the CT study centered on the brain (exam duration: 4 min, X-Ray beam duration: 90 s, kVp: 40, current: 400 μA, rotation time: 60 s, angular views: 960). At the end of the CT acquisition, the bed with immobilized animal was removed and inserted in the β-Cube PET scanner for a 20 min static acquisition. During the exam, mice were maintained under anesthesia and body temperature and respiratory rate were constantly monitored.

X-Cube^®^ has a spatial resolution of 0.05 mm and β-Cube^®^ has a spatial resolution less than 1 mm and a sensitivity greater than 10% over the field of view. CT and PET data were reconstructed using the proprietary Molecubes software included in the system. CT images were reconstructed with a 200 μm isotropic pixel size using a standard ISRA algorithm. PET images were reconstructed using a List-Mode Ordered Subset Expectation Maximization (OSEM) algorithm with 30 iterations and 400 μm isotropic voxel size. Tracer decay, random coincidences, well counter and detector dead time corrections were applied. No post-reconstruction filtering was applied. Thanks to the bed positioning system, CT and PET images were automatically co-registered.

Twelve C57BL/6JRj male mice underwent MR study using a 7 T preclinical MR scanner (Bruker, BioSpec 70/30 USR, Paravision 5.0) equipped with 450/675 mT/m gradients (slew-rate: 3400–4500 T/m/s; rise-time: 140 µs). Mice were imaged under gas anesthesia (1.5–2% isoflurane in oxygen) in prone position with brain centered in the Field of View (FOV). During the examination, breathing rate and body temperature were monitored (SA Instruments, Inc.) and maintained stable around 40 breaths per minute and 37 °C, respectively. MR protocol included Turbo spin-echo 2D T2-weighted images (TR = 3400 ms, TE = 56 ms, FOV = 14 × 15 mm, thickness = 0.6 mm, matrix size = 256 × 160, averages = 9) and 3D T2-weighted sequences (TR = 2350 ms, TE = 54 ms, FOV = 14 × 15 mm, matrix size = 256 × 96 × 102 pixel, resolution = 0.118 × 0.118 × 0.118 mm^3^, averages = 1).

### Application of the brain template on the MPTP subacute mouse model: images acquisition and reconstruction

Three-month-old C57BL/6J male mice (n = 6, breeding of the University of Ferrara, Italy) were daily treated with MPTP (30 mg/kg i.p., in saline) for 7 days, then subjected to PET–CT with the TSPO-specific radioligand [^18^F]VC701 in a test–retest study. The first acquisition was made before the onset (d0) and the other at the end of MPTP treatment (d7).

The radiotracer [^18^F]VC701 was prepared as previously described^15^ and injected i.v. under feeding conditions (mean injected activity: 4.7 ± 0.3 MBq). CT and PET acquisitions were performed 100 min post injection using X-Cube and β-Cube, respectively. Each animal was anesthetized with 2% isoflurane in medical air and then positioned prone on the scanner bed for the CT scan centered on the brain (exam duration: 4 min, X-Ray beam duration: 90 s, kVp: 40, current: 400 μA, rotation time: 60 s, angular views: 960). At the end of CT acquisition, the bed with the immobilized animal was removed and inserted in the PET scanner for a 20 min static acquisition. During the exam, mice were maintained under anesthesia, body temperature and respiratory rate were constantly monitored. CT and PET data were reconstructed and co-registered as described above. Images were transformed voxel by voxel in Standard Uptake Values (SUVs) and scaled to the global mean uptake value to measure modifications in radioactivity concentration between conditions. PMOD VOIs template was applied as previously described^16^.

### New template creation

**Figure 1:**
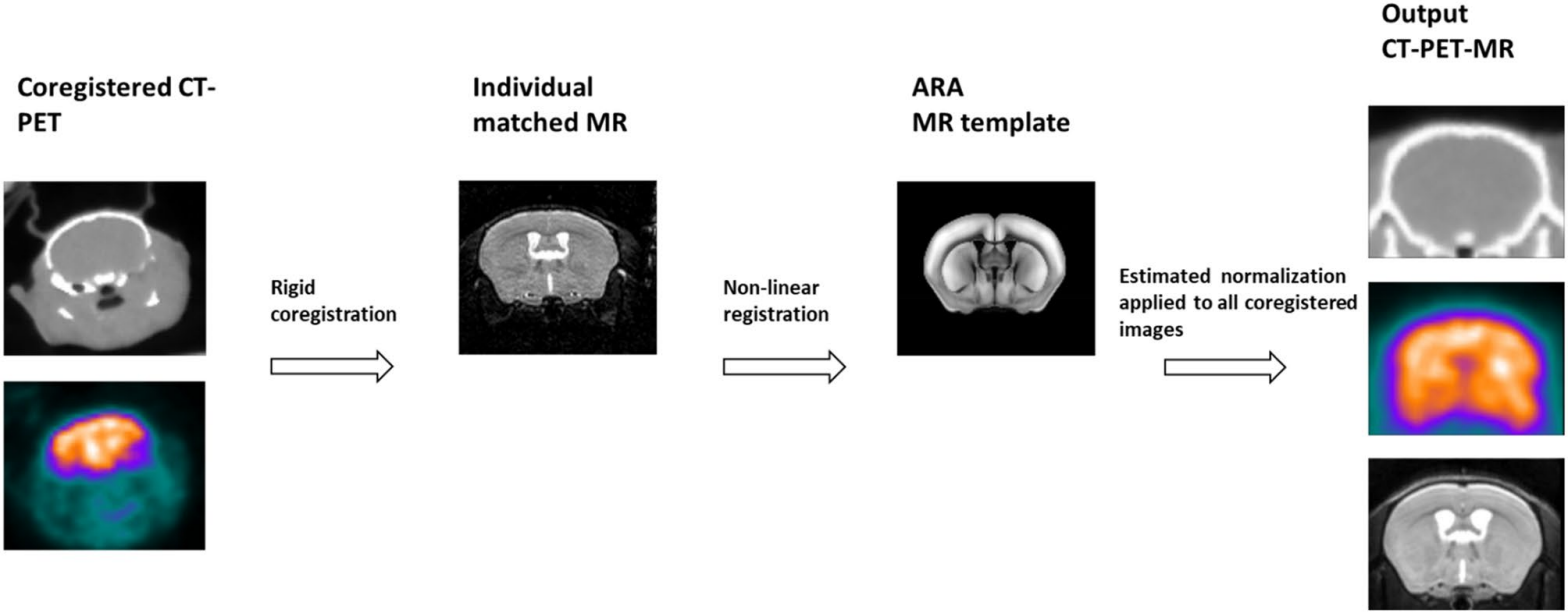
Schematic representation of the template creation starting from the acquisition of MR and [^18^F]FDG CT-PET images in healthy C57BL/6J male mice (n = 12 and n = 20, respectively). Images were normalized to the ARA MR template.

For each animal, CT and PET images were intrinsically co-registered (PET–CT) using the same bed in a single session, i.e. without moving the animal between the two (CT and PET) studies, while MR images were rigidly co-registered to PET–CT images using SPM-12 (https://www.fil.ion.ucl.ac.uk/spm/software/). Co-registered MR images from each animal were elastically deformed to the ARA using the multi-modal registration tool of the ANTsPy package, then averaged to create an MR template. Figure 1 summarizes the main steps involved in template generation. ARA has a relatively small bounding box (11.4 × 8 × 13.2 mm^3^) and does not include any extra-cerebral structures. With the purpose to properly define the limits of the brain, we used a larger bounding box (12.1 × 9.6 × 16.1 mm^3^) characterized by pixels dimension of 0.1 mm. After that, each MR image was spatially normalized using the SPM-12 “old normalization” toolbox, using the previously generated average image. This toolbox provides a regularized elastic intra-modality registration using a sum of squared differences as a target metric. This strategy is expected to maximize the robustness of the method. The obtained images were averaged to obtain the final MR atlas. Atlas was created in the same bounding box, although the isotropic voxels were resized to 0.2 mm due to the poor resolution of the modalities.

Alignments between the result and the original atlas were visually assessed by imaging experts, using anatomical landmarks.

CT and PET atlases were generated by averaging images that were spatially normalized using the deformation found on the MR image.

### Validation of the proposed registration algorithms

By using the 3D MR-based normalization as “gold standard”, we compared the effectiveness of the CT-based normalization pipeline. Due to the lack of cortical gyrification in mice, we hypothesized that the registration quality, based on the bone structures and soft tissue, would be sufficient even if internal structures are not visible in CT. For this evaluation, we normalized the images using both a CT-based and the MR-based pipeline and measured the point-wise distance in the deformation field. The deformation error was quantified using the Root Mean Squared Displacement. A pixel at a location *x’* in the template space can correspond to either the pixel *x*_*CT*_ when the CT based deformation is applied, or to the pixel *x*_*MR*_ when the MR-based one is used. All are 3 dimensional position vectors. We define the displacement between the two deformations in every pixel *x*′ as $\lVert x_{CT} - x_{MR}\rVert$. The root mean squared displacement over all subjects for one pixel is then $\sum\nolimits_{i} {\sqrt {\left\| {x_{{CT}} - x_{{MR}}} \right\|^{2}} }$.

### Regions definition

A template image (http://download.alleninstitute.org/informatics-archive/current-release/mouse_ccf/average_template/) and the VOIs definitions (http://download.alleninstitute.org/informatics-archive/current-release/mouse_ccf/annotation/ccf_2017/) were downloaded. The version with 100 µm isotropic voxels was selected. From the ARA ontology (http://help.brain-map.org/display/api/Atlas+Drawings+and+Ontologies), VOIs were grouped and selected according to the following criteria:

- Large compared to PET resolution (> 1.5 mm^3^)
- Enough regions to map the whole brain
- Representative of the main grey matter anatomical areas and according to the “Brain – Major Division” of the ARA.

After defining the list of VOIs, the actual contour was created by including every substructure as defined by ARA. To achieve a more regular shape, a one voxel-erosion operation was applied to each VOI.

### Application of the brain template to the MPTP subacute mouse model: template-based analysis

The new template developed for PET–CT images analysis purpose was applied in the study of the brain of a PD mouse model in order to improve the analysis of the areas involved in microglia/macrophage activation. PET–CT images of the mice undergoing MPTP treatment were analysed using the proposed pipeline, namely warping to the ARA space using the CT template, and radioactivity extraction in the previously defined VOIs. As for comparison, the analysis was also performed using the VOIs definitions included in the PMOD software, based on the studies of Ma and Mirrione^12,17^. PET–CT images were co-registered to this atlas, and radioactivity intensity was extracted using the VOIs included in this package. Table 1 reports the content of the provided toolbox and the suggested use of each component.

**Table 1:** Content of mouse brain template generated for PET–CT image analysis with description of each step and application.

Package content	Description	Intended use
Allen Reference Atlas—Nifti	ARA converted to nifti format	Facilitate comparison of coordinates between in-vivo neuroimaging and ARA
MR template	Mean of 12 high-res 3D-T2 MR scans of healthy mice, aligned to ARA	Reference template for spatial normalization of mice MR images
CT template	Mean of 12 CT scans of healthy mice, aligned to ARA	Reference template for the CT-based normalization pipeline
FDG-PET template	Mean of 12 FDG-PET scans of healthy mice, aligned to ARA	Could be used for PET-based normalization pipelines. CT-based ones are encouraged however
ROI maps	Labelled image of 27 ROIs in ARA space	To be used for the analysis of images after alignment in standard space
Co-registration batch script example	Batch file for SPM-based rigid registration	File containing all the optimized settings to run a rigid co-registration in SPM. Optimized for mice brains
Normalization batch script example	Batch file for SPM-based elastic normalization	File containing all the optimized settings to run the elastic deformation in SPM. Optimized for mice brains

### Statistical analysis

In the MPTP experiment, [^18^F]VC701 activity values were extracted from each volume of interest before and after 7 days of treatment. Radioactivity values were then scaled by the cerebral global mean. Finally, neuroinflammation, measured as the increase of [^18^F]VC701 uptake at day 7 compared to day 0, was assessed in each brain region using the Student’s t test for paired samples. FDR adjustment was applied to account for multiple comparisons.

## Results

### Regions definition and template creation

**Figure 2:**
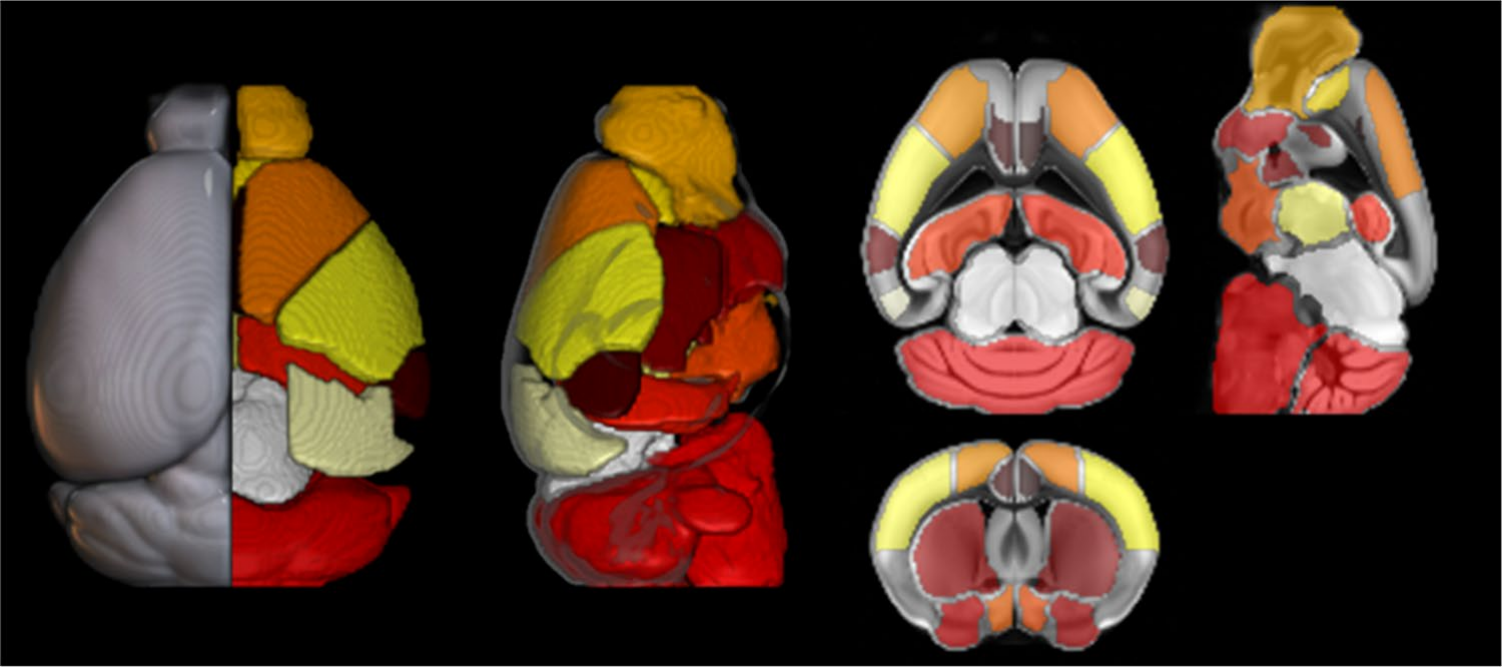
Representation of the VOIs defined in this work. The VOIs are superimposed to the ARA MR volume. Left: Dorsal 3D rendering; Center: Lateral 3D rendering; Right: axial, coronal and sagittal cross-sections.

The twenty-seven regions obtained are shown projected on reference images in Fig. 2.

**Figure 3:**
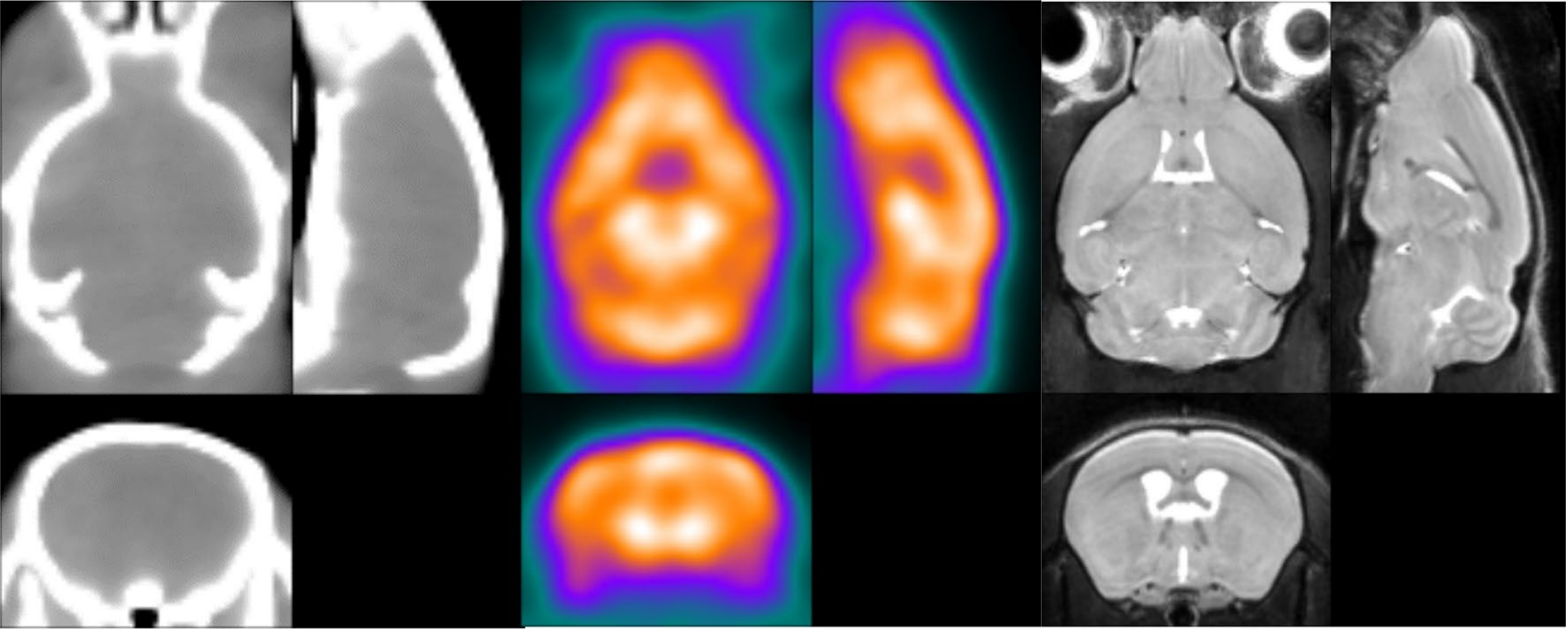
Three-plane view of the CT (right), PET (center) and MR (right) image templates obtained with the proposed normalization method, aligned to the ARA.

These regions include cerebral cortex, cerebellum, left/right dorsal and left/right ventral basal ganglia, thalamus and midbrain, as listed in Table 2. As stated above, these regions cover the anatomical structures of the whole brain starting from ARA macro-regions and were defined according to the spatial resolution of the PET–CT scanner (VOI range: 1.7 to 53 mm^3^). An imaging expert reviewed the images to visually assess the congruence between the VOIs and the regions defined on the registered MR scans. An example of the resulting partition is shown in Fig. 2. Figure 3 shows the CT, MR and PET templates produced.

**Table 2:** List of structures and abbreviations included in the template (L/R = both left and right) with each volume indicated.

Name	Abbreviation	Laterality	Volume (for each side) (mm^3^)
Anterior cingulate area	ACA	L/R	1.7
Auditory areas	AUD	L/R	1.9
Cerebellum	CB	Central	53.3
Hindbrain	HB	Central	47.1
Hippocampal region	HIP	L/R	8.9
Hypothalamus	HY	L/R	5.7
Midbrain	MB	Central	26.7
Olfactory areas	OLF	L/R	9.5
Orbital areas	ORB	L/R	11.3
Somatomotor areas	MO	L/R	2.0
Somatosensory areas	SS	L/R	13.2
Striatum dorsal	STRd	L/R	12.0
Striatum ventral	STRv	L/R	4.6
Thalamus	TH	L/R	8.2
Visual areas	VIS	L/R	4.8

### Validation of the CT-based registration pipeline

The root mean squared displacement observed between the CT-based registration and the MR-based registration was small. The global average observed was 0.43 mm, with minimum (0.339 mm) in the dorsal right striatum and maximum (0.630 mm) in the left auditory cortex (Table 3).

**Table 3:** Displacement between regional volume (mm) found in the new VOIs template compared to the ARA MR template.

Area	Root mean squared displacement (mm)
Anterior cingulate area left	0.359
Anterior cingulate area right	0.344
Auditory areas left	0.546
Auditory areas right	0.630
Striatum dorsal left	0.379
Striatum dorsal right	0.339
Striatum ventral left	0.417
Striatum ventral right	0.393
Cerebellum	0.549
Hindbrain	0.503
Hippocampal region left	0.397
Hippocampal region right	0.488
Hypothalamus left	0.375
Hypothalamus right	0.380
Midbrain	0.477
Olfactory areas left	0.392
Olfactory areas right	0.386
Orbital areas left	0.402
Orbital areas right	0.346
Somatomotor areas left	0.371
Somatomotor areas right	0.365
Somatosensory areas left	0.407
Somatosensory areas right	0.477
Thalamus left	0.353
Thalamus right	0.395
Visual areas left	0.495
Visual areas right	0.656

### Results of [^18^F]VC701 PET–CT in the MPTP subacute mouse model

**Figure 4:**
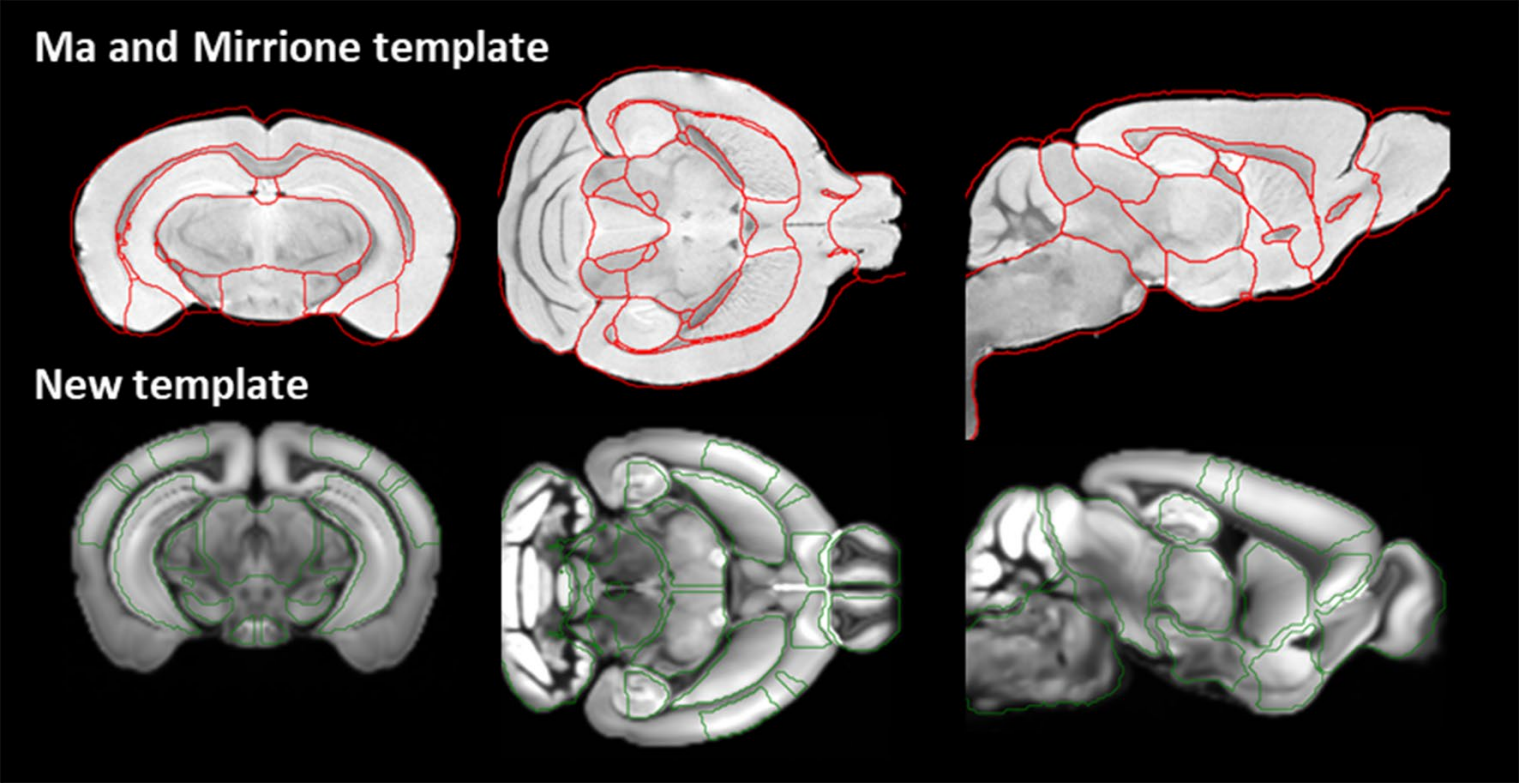
Visual comparison between Ma and Mirrione template in red (top) and the new brain template in green (bottom).

The application of the new VOIs template in the SPM analysis of the [^18^F]VC701 PET–CT images in PD animals is able to identify which sub-regions are involved in microglial/macrophage activation (Table 4) in comparison to the macro-regions identified using the PMOD atlas. Figure 4 shows the two templates applied. In particular, the neurotoxin administration induced a specific increase of radioligand uptake in 11 sub-regions, including left and right dorsal striatum (*p* < 0.001), left (*p* < 0.001) and right (*p* < 0.05) thalamus, left (*p* < 0.05) and right (*p* < 0.01) orbital areas, right somatomotor and somatosensory cortex (*p* < 0.05) and midbrain (*p* < 0.05). The PMOD template, instead, identified significant radiotracer increase in right and left striatum and thalamus (*p* < 0.001) and in central gray region (a part of the midbrain) (*p* < 0.05) but not in the cortex, possibly due to the lack of areas partition. After false discovery rate adjustment, 6 sub-regions remained significant with the proposed method, while only 3 survived with the PMOD atlas analysis.

**Table 4:** T-test significance (*p* values) of regional increase of the [^18^F]VC701 radioligand uptake after MPTP treatment, measured using the new template (left) and the PMOD template (right).

Area of the new atlas	*p* value	Area of Ma and Mirrione template	*p* value
Anterior cingulate area left	0.0068**^†^	Amygdala R	0.7648
Anterior cingulate area right	0.0039**^†^	Amygdala L	0.6727
Auditory cortex left	0.5459	Cortex	0.0730
Auditory cortex right	0.4974		
Striatum dorsal left	0.0007***^†^	Striatum R	0.0003***^†^
Striatum dorsal right	0.0006***^†^	Striatum L	0.0002***^†^
Striatum ventral left	0.7122		
Striatum ventral right	0.9702		
Cerebellum	0.9875	Cerebellum	0.9992
Hindbrain	0.4002	Basal forebrain septum	0.1229
Hippocampal region left	0.1113	Hippocampus L	0.3103
Hippocampal region right	0.0801	Hippocampus R	0.5574
Hypothalamus left	0.2758	Hypothalamus	0.5217
Hypothalamus right	0.7514		
Olfactory areas left	0.7972	Olfactory bulb	0.2139
Olfactory areas right	0.6618	Superior colliculi	0.1677
Orbital areas left	0.0311*	Inferior colliculi R	0.8842
Orbital areas right	0.0055**^†^	Inferior colliculi L	0.5472
Somatomotor areas left	0.0634		
Somatomotor areas right	0.0392*		
Somatosensory areas left	0.6806	Brain stem	0.9439
Somatosensory areas right	0.0271*		
Thalamus left	0.0003***^†^	Thalamus	0.0040**^†^
Thalamus right	0.0242*		
Visual areas left	0.8395	Central gray	0.0148*
Visual areas right	0.0889	Midbrain R	0.3966
Midbrain	0.0436*	Midbrain L	0.4016

*p < 0.05; **p < 0.01; ***p < 0.001.

^†^Significant after FDR adjustment.

**Figure 5:**
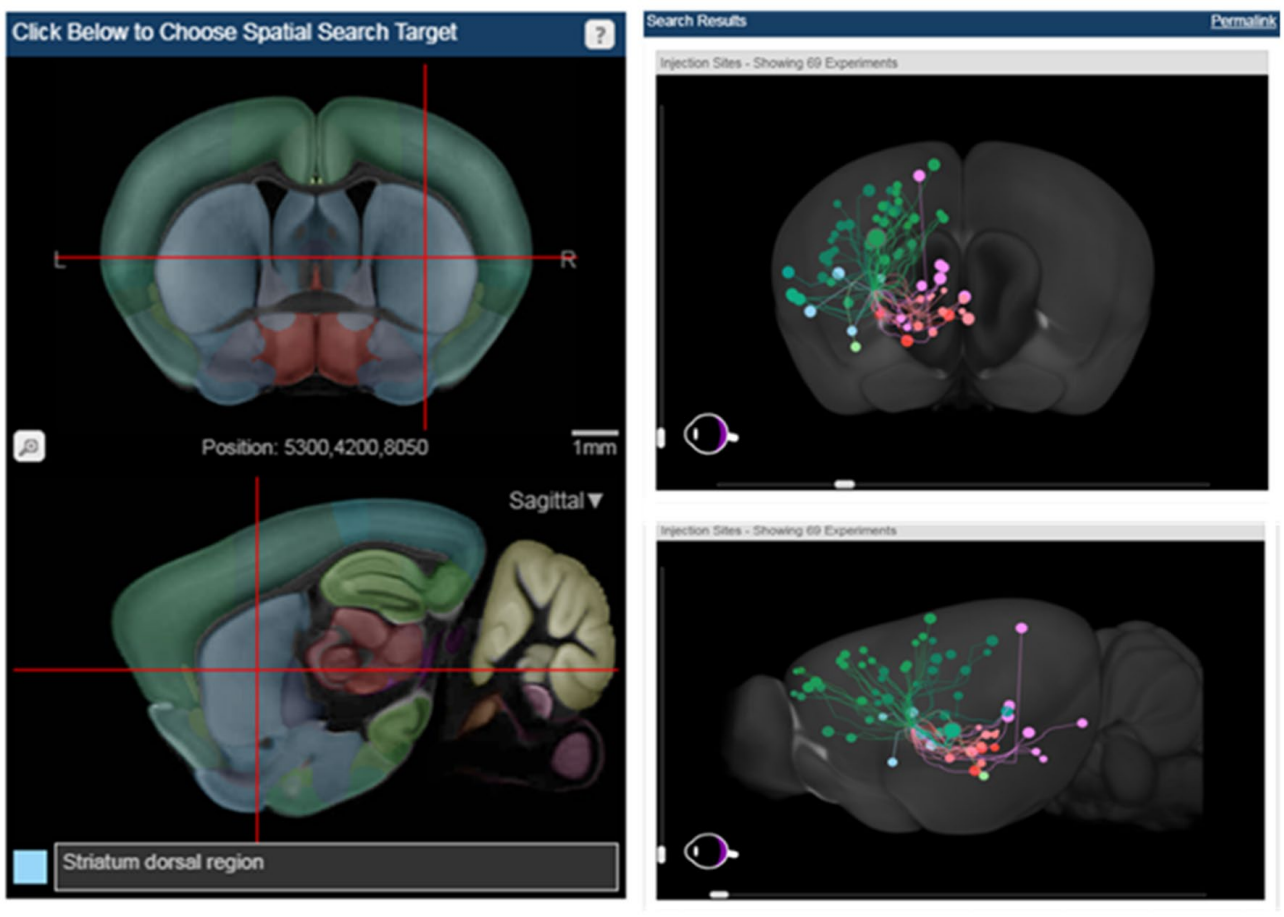
High-resolution map of neural connections of dorsal region of striatum in the mouse brain, built on an array of mice genetically engineered to target specific cell types. The colored dots indicate the position of the regions connected to the selected one: green for motor areas, dark green for somatosensory areas, pink for substantia nigra, orange and red for thalamic nuclei. Image credit: Allen Institute (www.alleninstitute.org).

In addition, striatal subdivision showed that the inflammatory reaction was limited to the dorsal sub-region. This is in line with the information contained in the ARA connectivity tool (https://connectivity.brain-map.org/projection) developed by Allen group in the same space. This tool identifies functionally connected areas based on an array of mice genetically engineered to target specific cell types. By using the “Spatial Search” option of the tool, it is possible to find all the regions connected in a circuit starting from a source structure. By filtering for the dorsal part of the striatum, we observed a connection with all the cortical and subcortical regions in which we found a significant increase of [^18^F]VC701 (Fig. 5 and Table 3). Conversely, by filtering for the ventral striatum, a region unaffected by MPTP, we found connections with other brain regions equally unaffected by the toxin (Supplementary Fig. 1). This confirmed the overlap of PET and connectivity data.

## Discussion and conclusions

The development of brain templates enables the precise identification of the elements (sub-regions) that are fundamental for the study of pathology progression and response to treatment in neurodegeneration and neuroinflammation. Three-dimensional brain atlases for small rodents are available to study neuroanatomy in relation to gene expression, cell types and functional pathways. One of the more advanced and complete for mouse is the Allen Brain Atlas (https://atlas.brain-map.org/) that allows a correlation of neuropathology with behaviour or phenotype. PD is characterized by loss of dopaminergic neurons and α-synuclein aggregation in the Substantia nigra pars compacta, a small region within the midbrain. A key and recently recognized event in PD pathogenesis is the neuroinflammatory process triggered by α-synuclein aggregation^18,19^. The fact that this process is a very early event during PD development is confirmed by different in vivo PET studies in prodromal PD patients^20,21^. The inflammatory response in PD is carried out by microglia and, to a minor extent, astrocytes. Activated microglia produce detrimental cytokines and inflammatory factors, and overexpress a variety of receptors as the translocator protein (TSPO). Using radiotracers specific for TSPO, as [^11^C]PK11195, it was possible to follow microglial activation over PD progression and find a negative correlation between the increase of neuroinflammation and dopamine content in midbrain^22^. Therefore, while MR imaging is applied to the study of neuroanatomical changes, which are particularly evident in advanced stage of PD, PET imaging is able to detect early biochemical modifications that are already present in pre-symptomatic/premotor phases. Image analysis plays a fundamental role in identifying dysfunctional regions and integrating information deriving from different sources. Different PD rodent models have been developed and studied using PET and radiotracers that allow the identification of biomarkers of disease development and progression^23^. In the preclinical setting, a major limitation is represented by the lack of standardization of image acquisition and tools for the analysis.

We developed a brain atlas for mouse that conforms to the CCFv3 and provides regions that are optimized for PET–CT imaging analysis and defined within its ontology. This tool enables to develop protocols independent of high resolution 3D anatomical MR, which can be costly and difficult to perform due to anaesthesia duration constraints. Despite of lower quality compared to MR-based registration, the average displacement measured was < 500 µm in most regions. This is significantly lower than PET spatial resolution in state-of-the art systems.

The present work has some limitations. A full study on the effect of the CT image quality on the image registration precision was not performed. However, it can be expected that any acquisition protocol with a good spatial resolution (e.g.: 200 µm^3^ pixel or smaller) and visualization of the cortical bone, should perform identically. In addition, this pipeline was not validated against animals with uncommon anatomies, such as after major surgeries to the skull or extreme degrees of atrophy.

When applied to the PD model, the template allowed the recognition of the sub-regions involved in response to a neuronal insult, such as subacute MPTP. In particular, we found that after 7 days of MPTP treatment, the uptake of the TSPO-specific radiotracer [^18^F]VC701 was significantly increased in dorsal part of striatum, thalamus and midbrain, a region including substantia nigra. These regions are directly affected by the neurodegenerative process (substantia nigra and striatum) or are involved in PD motor dysfunction (thalamus)^24,25^. In fact, we found a significant radiotracer uptake increase also in somatomotor, somatosensory, anterior cingulate and orbital cortex areas, which undergo structural and functional alteration during PD, as measured by MR and PET^26^.

Subacute MPTP^14^ increased the expression of different pro-inflammatory cytokines and induce microglia activation in different brain regions such as anterior cortex, striatum and midbrain^27–29^. We successfully measured this phenomenon in vivo with the TSPO-specific radioligand [^18^F]VC701, which has been already used by our group for the study of different neurodegeneration models^30,31^. The two areas with maximal elevation of [^18^F]VC701 uptake were striatum and thalamus, and were consistent between the two templates, even after FDR adjustment. The application of PMOD template failed to reveal significant changes of radiotracer uptake in cortical regions sampled as a whole, although microglial activation was observed in selected cortical areas such as orbital or cingulate cortex in PD^32^. In addition, the adaptation of the PET–CT volume to the ARA volume and the definition of anatomical regions that are superimposable offer the possibility to apply all the advanced tools developed for ARA, such as the connectivity tool (https://connectivity.brain-map.org/projection) that allows to reconstruct the different neuronal circuits within the brain. Therefore, when we selected the dorsal part of the striatum using this tool, we observed that this region is connected to cortical and subcortical areas in which we observed [^18^F]VC701 uptake increase, suggesting that the whole circuit was affected by the neurotoxin.

In mouse brain, cell body of dopaminergic neurons are mainly localized in three distinct subcortical regions: substantia nigra (A9 region), ventral tegmental area (A10) and retrorubral field^33^. The A9 region that projects to the dorsal part of the striatum is more susceptible to MPTP administration in comparison with A10 that, instead, projects to the ventral part of striatum^34^. In line with these findings, we found no neuro-inflammatory response in the ventral part of the striatum and other limbic cortical and subcortical regions.

As expected, when the connectivity tool was applied to the ventral part of the striatum we failed to find any association with brain regions with increased levels of radioligand. This example shows the value of the ARA association with Allen connectivity tools in the evaluation of the regional distribution of PET radiopharmaceuticals or interpretation of brain metabolic studies.

Moreover, different tools for gene expression have been developed and adapted to the ARA, increasing the possibility of integrating omics information for a given phenomenon^35^.

In conclusion, the brain template developed here represents an efficient tool for mouse PET–CT image quantification, making MR acquisition unnecessary and offering the unique advantage to merge molecular or structural data deriving from in vivo preclinical imaging with genetic, molecular and cell information present in ARA tools.

### Information sharing statement

The same animals and their PET–CT datasets were used for the preparation of the following articles: “In vivo susceptibility to energy failure parkinsonism and LRRK2 kinase activity” by Salvatore Novello and colleagues^16^.

## Supplementary Information

Supplementary Information.

## Data Availability

The datasets generated during and/or analysed during the current study are available from the corresponding author on reasonable request. The whole package will be made freely available for download on the NITRC repository when the paper will be published.
