# Supplementary material for: Development of a new toolbox for mouse PET–CT brain image analysis fully based on CT images and validation in a PD mouse model
Source: Sci Rep. 2022 Sep 22;12:15822. doi: 10.1038/s41598-022-19872-4 (PMC9500043; doi:10.1038/s41598-022-19872-4)
Supplement: Supplementary file 1 — Supplementary Information. [file 41598_2022_19872_MOESM1_ESM.docx]

**Supplementary Information**

**Development of a new toolbox for mouse PET-CT brain image analysis fully based on CT images and validation in a PD mouse model**

Presotto L^1,2^, Bettinardi V^1^, Mercatelli D^3^, Picchio M^1,4^, Morari M^3^, Moresco RM^1,2,5,6^and Belloli S^1,2,6^

^1^Nuclear Medicine Department, IRCCS San Raffaele Scientific Institute, Milan, Italy;

^2^Milan Centre for Neuroscience, University of Milano - Bicocca, Milan, Italy;

^3^Department of Neuroscience and Rehabilitation, University of Ferrara, Ferrara, Italy;

^4^Vita-Salute San Raffaele University, Milan, Italy;

^5^Medicine and Surgery Department, University of Milano - Bicocca, Monza (MB), Italy;

^6^Institute of Molecular Bioimaging and Physiology (IBFM) of CNR, Segrate (MI), Italy.

Corresponding author: Belloli Sara, Institute of Molecular Bioimaging and Physiology (IBFM) of National Research Council (CNR), Segrate (MI), Italy. e-mail address: [sara.belloli@ibfm.cnr.it](mailto:sara.belloli@ibfm.cnr.it); [belloli.sara@hsr.it](mailto:belloli.sara@hsr.it); phone: +39 02 26433640.


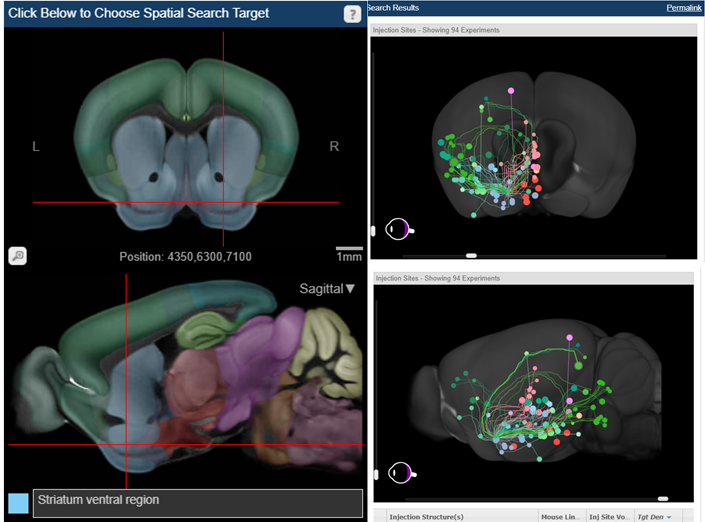


**Supplementary Figure 1.**

High-resolution map of neural connections of striatum ventral region in the mouse brain, built on an array of mice genetically engineered to target specific cell types. The coloured dots indicate the position of the regions connected to the selected one: light blue and light green for amygdalar nuclei, green for entorhinal areas, dark green for orbital areas and orange for mammillary nucleus.

Image credit: Allen Institute (www.alleninstitute.org).
